# Occult retinal neovascularization following intravitreal bevacizumab and laser treatment for retinopathy of prematurity

**DOI:** 10.1186/s12887-024-04784-1

**Published:** 2024-05-04

**Authors:** Wei Loon Ng, Adisak Trinavarat, La-ongsri Atchaneeyasakul

**Affiliations:** Department of Ophthalmology, Faculty of Medicine Siriraj Hospital, Mahidol University, 2 Wanglang Road, Bangkok, 10700 Thailand

**Keywords:** Retinopathy of prematurity, Plus disease, Occult neovascularization

## Abstract

**Background:**

We present a patient with retinopathy of prematurity (ROP) who developed worsening plus disease after complete regression of stage 3 ROP. The use of fundus fluorescein angiography (FFA) aided the visualization of occult neovascularization that caused the disease progression.

**Case presentation:**

The patient was at high risk for ROP due to low birth weight of 690 g and gestational age of 25 weeks. After the diagnosis of stage 3 ROP in zone I without plus disease, she was treated initially with bilateral intravitreal bevacizumab (IVB) and followed by laser photocoagulation 5 weeks later. Despite the resolution of ROP stage, the plus disease worsened. Neither systemic risk factors nor skip laser areas were observed. Hence, FFA was performed and subsequently identified occult neovascularization with active leakage. Additional IVB and laser treatment in the capillary dropout area inside vascularized retina were added. The plus disease improved but mild arteriolar tortuosity persisted.

**Conclusions:**

Worsening of plus disease after completion of laser ablation and IVB with complete regression of stage 3 ROP is rare. Systemic risk factors such as continuous oxygen therapy and cardiovascular disease should be ruled out. FFA aided in identifying occult neovascularization and prompted further treatment.

## Background

Retinopathy of prematurity (ROP) is a sight-threatening condition that retinal vascular formation was disrupted in premature infants with low birth weight. The incidence of ROP has been greatly reduced with advances in neonatal medical care [1]. ROP treatment protocol was guided by Early Treatment of Retinopathy of Prematurity (ETROP) [2].

Plus disease is a condition of arteriolar tortuosity and venous dilatation within the posterior pole based on the standard published photographs [3]. They are the indicators of disease activity and hence warrant treatment when the criteria are fulfilled. It can be accompanied by corneal haze, poor pupillary dilatation and vitreous haze. Typically, after successful ROP treatment, plus disease will improve followed by the regression of ROP stages. For those patients with persistent retinal vascular tortuosity and dilatation, cardiovascular disease should be investigated [4].

We present a patient with ROP who developed worsening of plus disease after complete regression of type I ROP following intravitreal anti-vascular endothelial growth factor (anti-VEGF) injection and laser photocoagulation. Since no systemic risk factors were identified, the fundus fluorescein angiography (FFA) was performed and revealed occult neovascularization that represents continuing disease progression.

## Case presentation

This infant was born via transvaginal delivery at gestational age of 25 weeks and the birth weight was 690 g. She received a total of 116 days of oxygen supplementation. She was diagnosed with patent ductus arteriosus, which was later closed spontaneously, and anemia that did not require blood transfusion during the early postnatal period.

**Fig. 1:**
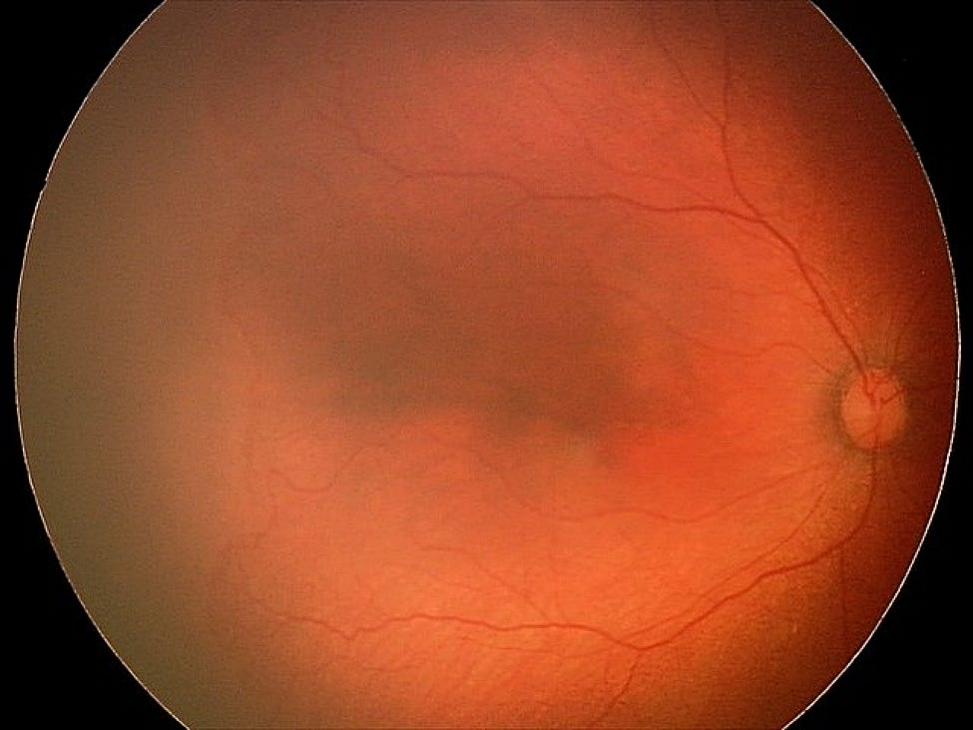
Fundus photo of the right eye before first intravitreal bevacizumab was given Legends : Stage 3, zone I, no plus

**Fig. 2:**
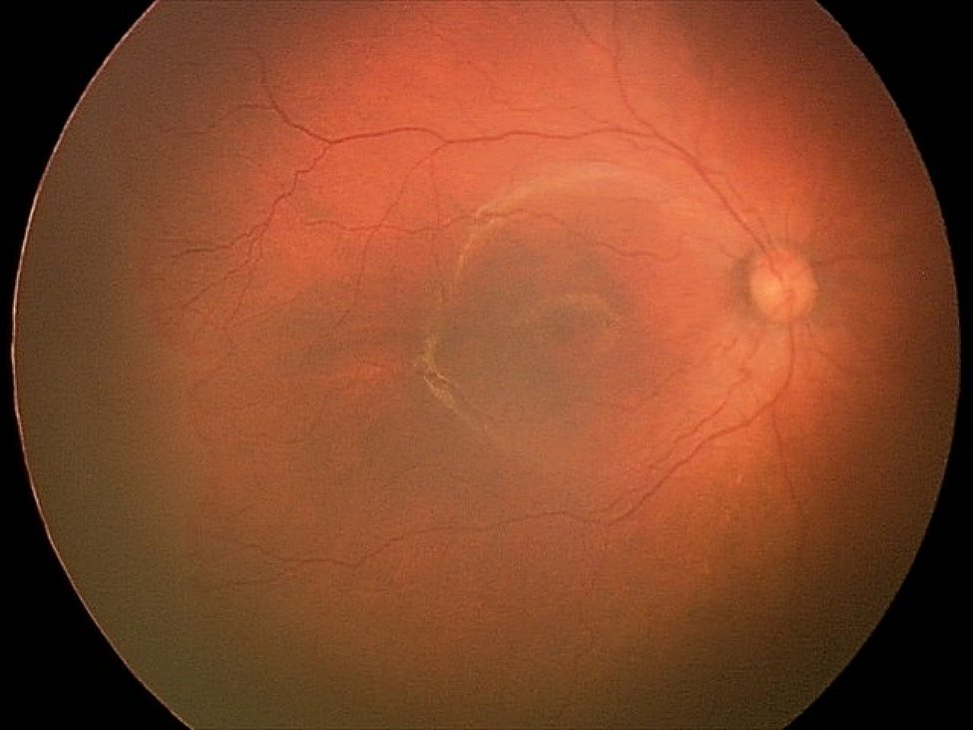
Fundus photo of the right eye before first session of laser was performed Legends : Stage 3, zone I with pre-plus

**Fig. 3:**
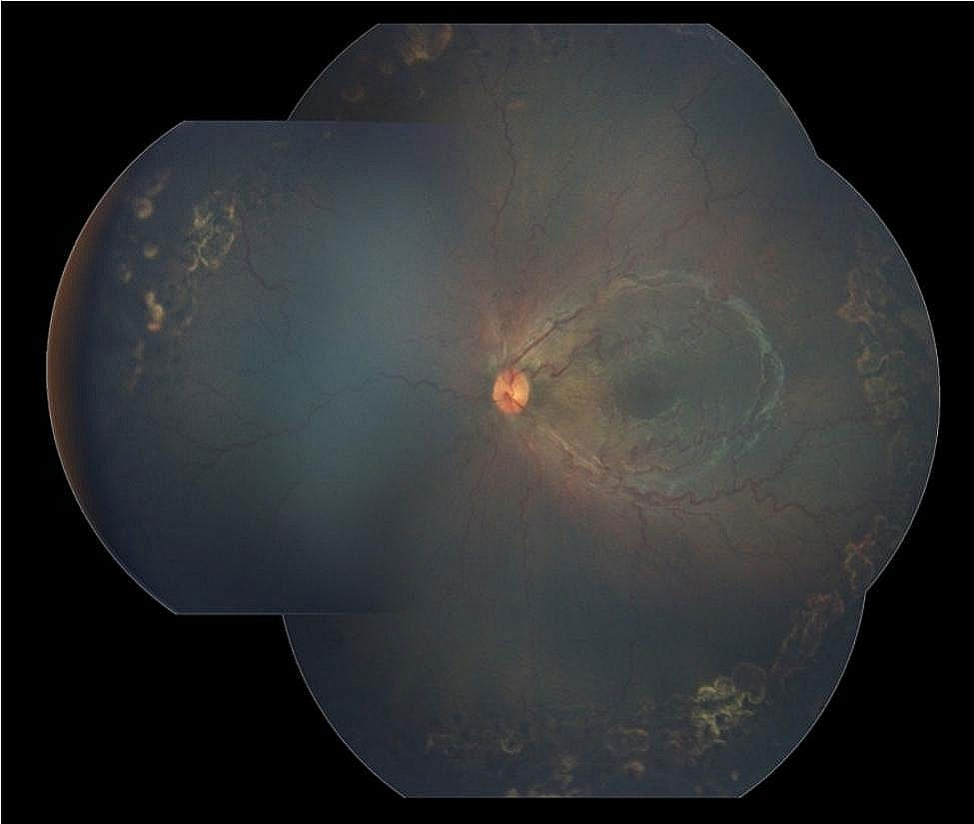
Fundus photo of the left eye Legends : ROP staging regression but worsening of plus disease after laser photocoagulation

**Fig. 4:**
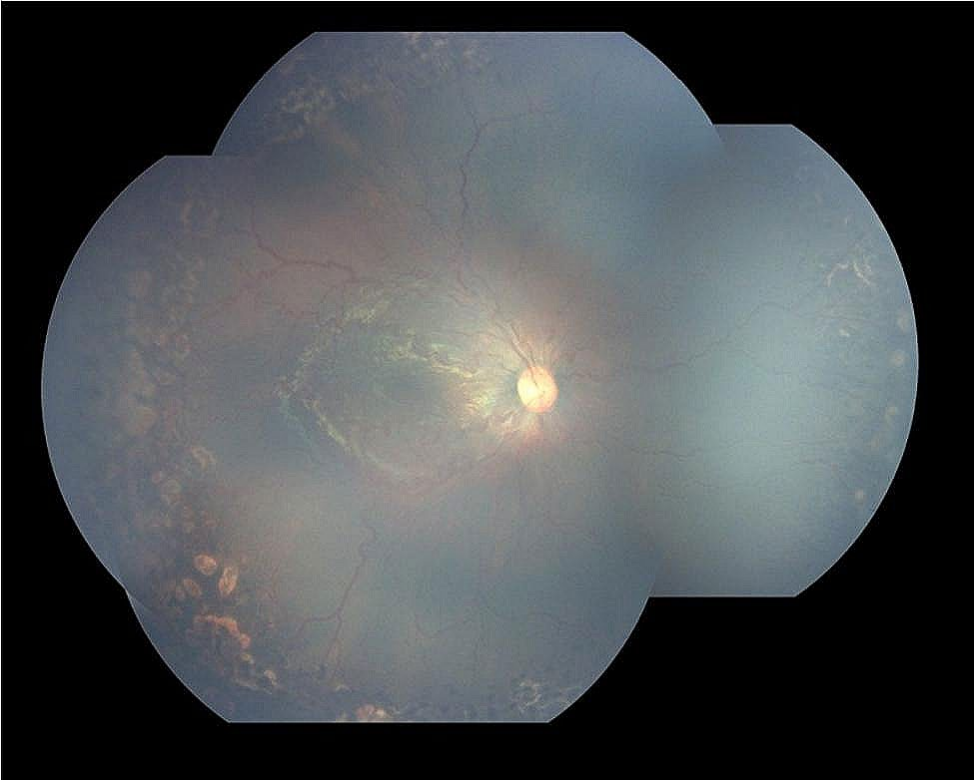
Fundus photo of the right eye Legends : ROP staging regression but worsening of plus disease after laser photocoagulation

This patient was first diagnosed with ROP stage 1, zone 1, without plus disease at 32 weeks post-menstrual age (PMA). She then progressed quickly to stage 3, zone 1, without plus disease (type 1 ROP) on the subsequent week (Fig. 1). The first dose of intravitreal bevacizumab 0.5 mg was given bilaterally. The ROP did not regress, and she received laser photocoagulation in both eyes 5 weeks after the injection. Upon laser treatment, the diagnosis was changed to stage 3, zone 2, with pre-plus disease bilaterally (Fig. 2). The ROP then regressed, but the vascular tortuosity and dilatation were worsening even though she had already been weaned off oxygen at PMA of 41 weeks and there was no skip area of laser treatment on either eye’s fundus (Figs. 3 and 4). Second session of laser treatment was given to both eyes posterior to laser scars at PMA of 47 weeks.

**Fig. 5:**
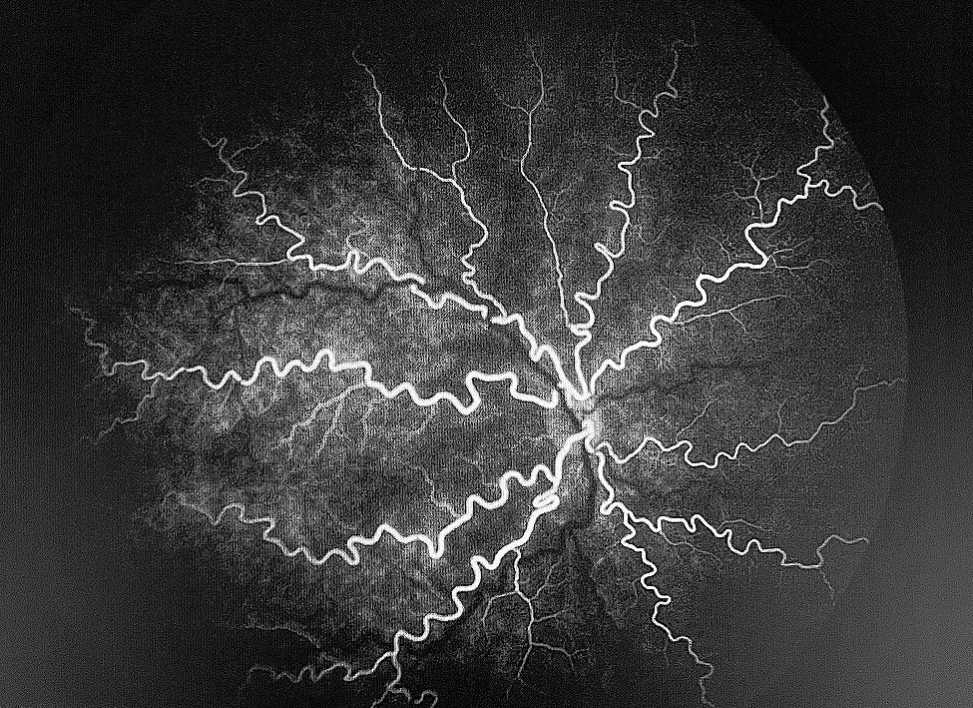
FFA at arterial phase (right eye) Legends : Arteriolar dilatation and tortuosity were noted in all four quadrants

**Fig. 6:**
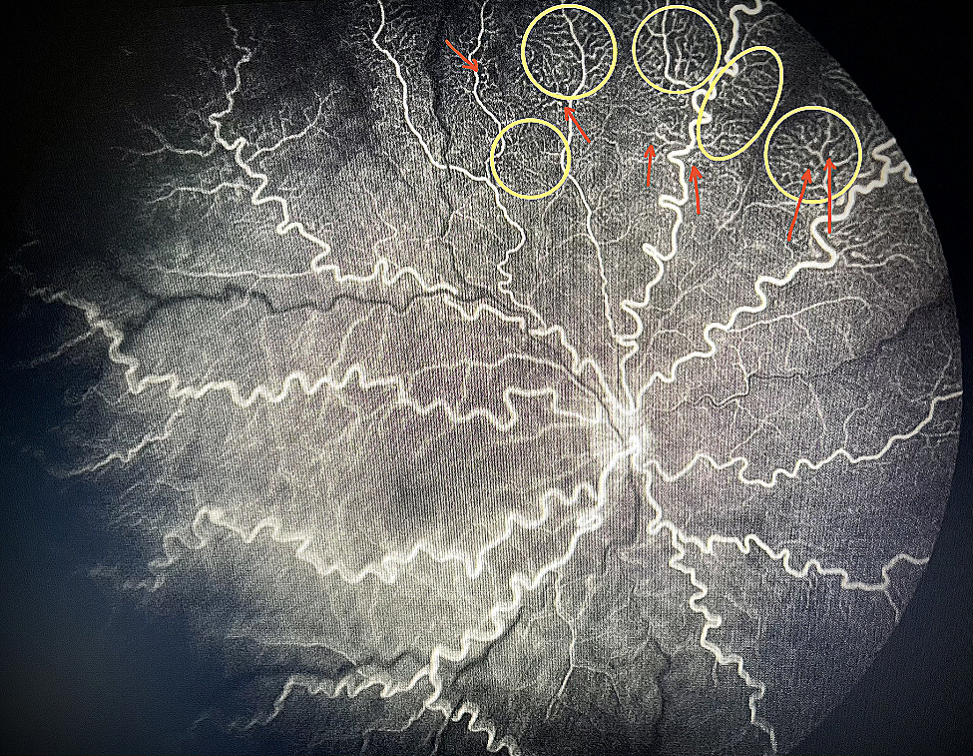
FFA at early venous phase (right eye) Legends : Abnormal branching of capillaries (yellow circle) with capillary terminal tuffs (red arrow) were noted. Abnormally vascular dilatation and tortuosity were present

**Fig. 7:**
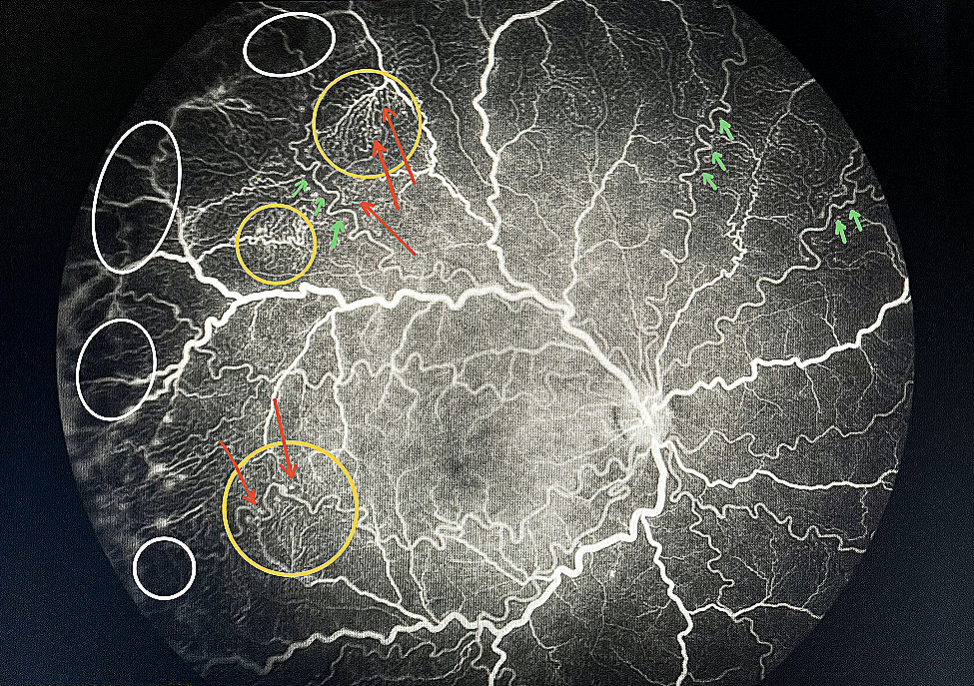
FFA at late venous phase (posterior pole of the right eye) Legends : The dilated veins and arteriolar tortuosity were demonstrated better in the FFA than fundus photos. Abnormal branching of capillaries (yellow circle) and capillary tuffs (red arrows) at their terminals were noted. There was laminar pattern of periarteriolar poor perfusion along the arterioles (green arrows). Peripheral leakage signified active disease. Capillary fallout areas were noted (white circles)

**Fig. 8:**
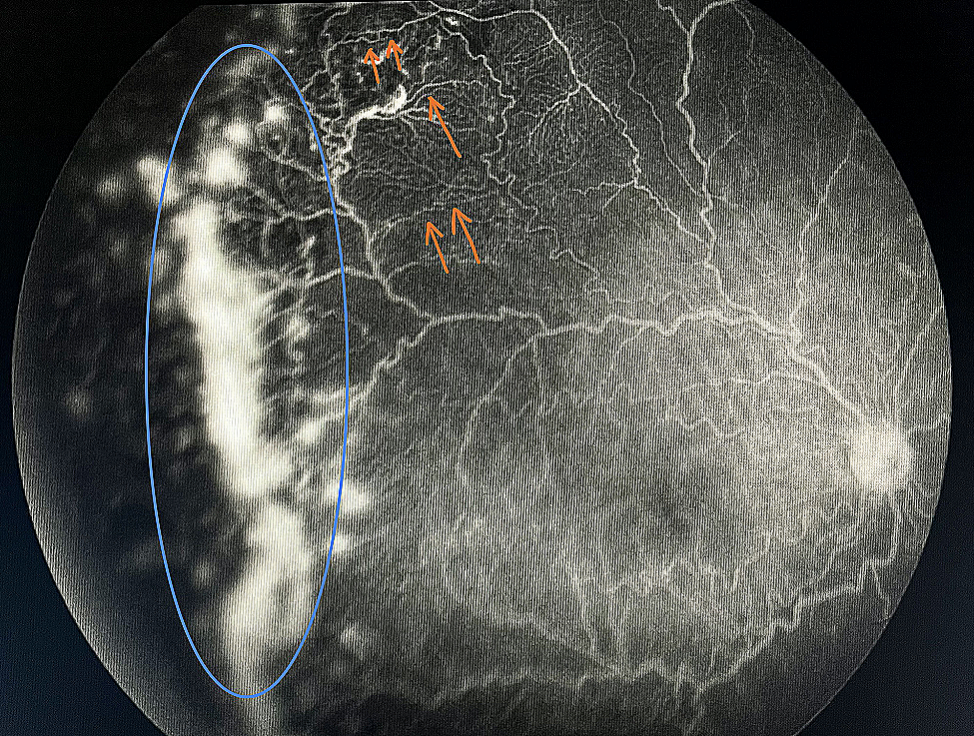
FFA at late venous phase (temporal periphery of the right eye) Legends : Worsening of vascular leakage at the peripheral vascular-avascular junction (blue circle). Abnormal arteriovenular shunts were noted (orange arrows)

**Fig. 9:**
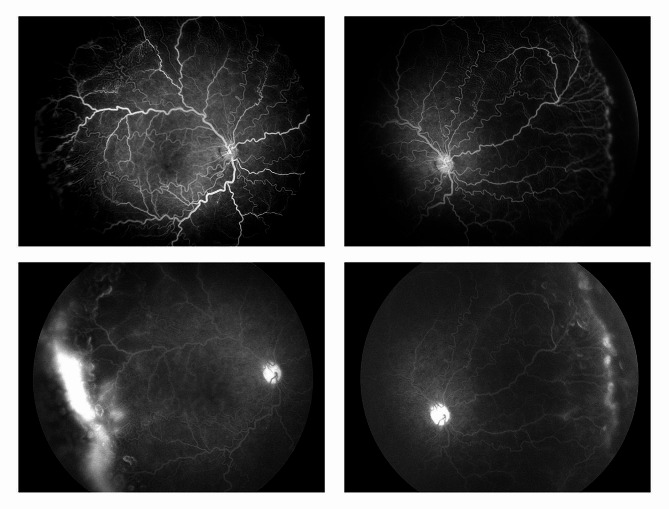
FFA photos during early phase of (upper two photos) and late phase (lower two photos) of both eyes Legends : The leakages worsened towards the late phase at vascular-avascular junction

**Fig. 10:**
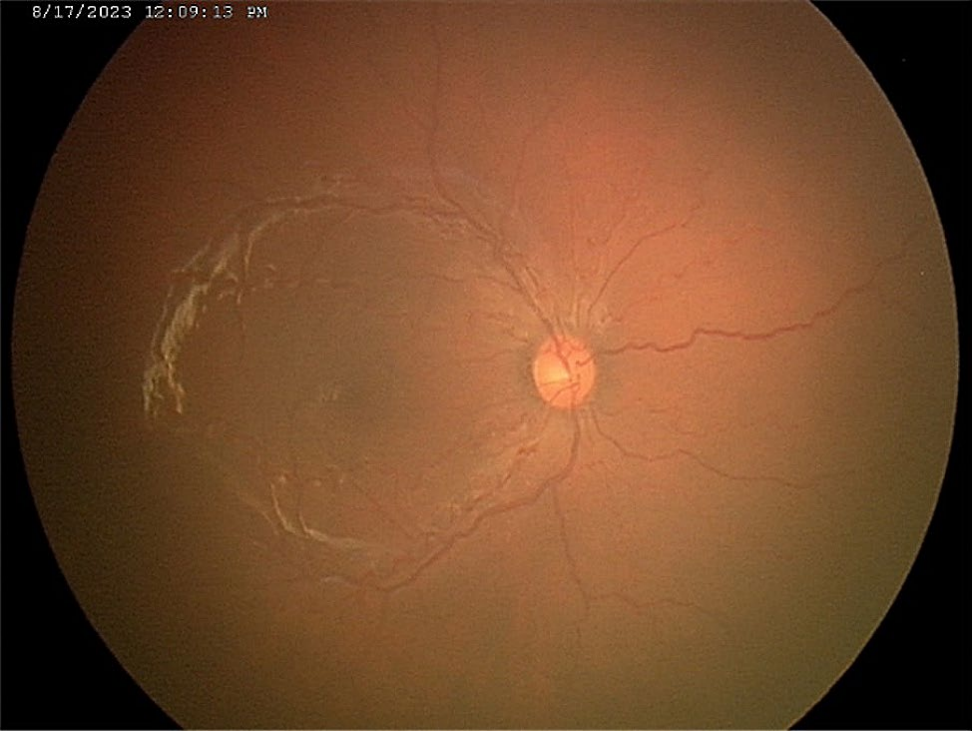
Fundus photo of the right eye at 60 weeks of PMA Legends : Complete ROP staging regression but mild vascular tortuosity persisted

**Fig. 11:**
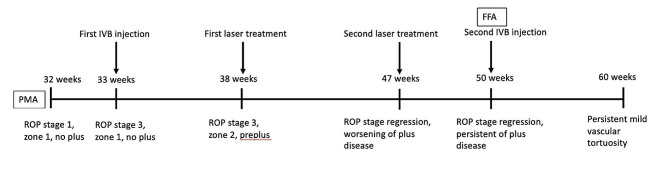
Summary of the course of disease and treatment Legends : Nil

Screening by a pediatric cardiologist showed no evolving cardiovascular disease or anemia. As the plus features were progressing, FFA was performed at the PMA of 50 weeks. It revealed the areas of leakage at the vascular-avascular junction, prominent abnormal capillary branches extending from major vessels, arterio-venous shunts at the periphery, and areas of capillary fallout (Figs. 5, 6, 7, 8 and 9). These were the indicators for further anti-VEGF injection and laser therapy. The patient then received the second dose of intravitreal bevacizumab. During the latest follow-up at 60 weeks of PMA, the ROP stages were completely regressed. Mild arteriolar tortuosity can still be observed (Fig. 10). The entire course of disease and treatment was summarized (Fig. 11).

## Discussion

According to the ETROP, treatment criteria have been indicated in type 1 ROP by means of laser photocoagulation [2]. Bevacizumab Eliminates the Angiogenic Threat of Retinopathy of Prematurity (BEAT-ROP) study and Ranibizumab versus Laser Therapy for the Treatment of Very Low Birthweight Infants with Retinopathy of Prematurity (RAINBOW) study had proven the role of anti-vascular endothelial growth factor in treating ROP, especially zone 1 and aggressive posterior ROP [5, 6].

In our case, there was no additional systemic risk factors identified during the course of ROP after treatment had been started. Despite complete regression of the ROP stages, the plus disease progressed, especially the vessel dilatation and tortuosity, which became more evident. This contrasts with the previous study, which found that regression of plus disease occurred earlier than regression of stage 3 ROP [7]. Yannis et al. reported a case of persistent plus disease after laser in ROP likely secondary to the tetralogy of Fallot [4]. In our case, there was no cardiovascular disease detected, and the reason for the worsening plus disease could not be explained.

FFA plays a role in the diagnosis and assessment of disease activity in pediatric vascular disorders such as familial exudative vitreoretinopathy, Coats disease, and ROP [8, 9]. FFA was not routinely performed in all cases of ROP despite its better role in diagnosing abnormalities in the retinal vasculature. It should be indicated only when the diagnosis is in doubt and to guide the treatment plan in atypical cases like ours.

In ROP, FFA could clearly demonstrate abnormal extraretinal vessels, occult neovascularization, and leakage [10]. In our case, the presence of leakages at the border of vascular-avascular areas indicated that the disease was still active. IVB could delay normal retinal vascularization while halting ROP activity. In this case, shunt vessels and areas of non-perfusion were seen. Massive fine meshworks of retinal vessels are seen extending from the major vessels.

## Conclusions

Progressive plus disease despite complete regression of ROP stages is rare and challenging. We highlighted the role of FFA in identifying the cause of persistent plus disease once the cardiovascular disease was ruled out.

## Data Availability

All the relevant patient information was obtained from the medical record system of Faculty of Medicine, Hospital Siriraj, Thailand.

## Abbreviations

Anti-VEGF: Anti-vascular endothelial growth factor
BEAT-ROP: Bevacizumab Eliminates the Angiogenic Threat of Retinopathy of Prematurity
ETROP: Early Treatment of Retinopathy of Prematurity
FFA: Fundus fluorescein angiography
IVB: Intravitreal bevacizumab
PMA: Post-menstrual age
RAINBOW: Ranibizumab versus Laser Therapy for the Treatment of Very Low Birthweight Infants with Retinopathy of Prematurity
ROP: Retinopathy of prematurity
